# Supplementary material for: Exploring the Willingness to Accept SARS-CoV-2 Vaccine in a University Population in Southern Italy, September to November 2020
Source: Vaccines (Basel). 2021 Mar 18;9(3):275. doi: 10.3390/vaccines9030275 (PMC8003195; doi:10.3390/vaccines9030275)
Supplement: Supplementary file 1 [file vaccines-09-00275-s001.pdf]

A. SOCIO-DEMOGRAPHIC, PROFESSIONAL AND ANAMNESTIC CHARACTERISTICS

The questions in this section are aimed to acquire information on your socio-demographic, professional and anamnestic characteristics

A1. Year of birth __________

A2. Gender □ Male □ Female

A3. What is your present marital status? □ Married □ Cohabitant □ Unmarried □ Other ______________________

A4. What is the highest education leaving certificate, diploma or education degree you have obtained?

□ None □ Primary □ Lower secondary □ Upper secondary □ Higher

A5. What is your professional role?

□ Faculty Member □ Student (Degree and year of course) ____

□ Administrative staff □ Other ____

A6. What is your current working area? _________________________________

A7. Do you have any chronic disease? □ No □ Yes, which one?

Chronic disease	How old were you when it was first diagnosed	Chronic disease	How old were you when it was first diagnosed

A8. Have you been tested positive for COVID-19? □ No □ Yes

A9. Have you been in close contact to anyone who has been tested positive for COVID-19?

□ No (go to A.10) □ Yes (more than one is allowed)

□ Household/cohabitant □ Relative not cohabitant □ Co-worker □ Friend □ Acquaintance □ Neighbour

A10. Have you experienced in the last ten months, any of the following symptoms? (more than one is allowed)

□ None □ Headache □ Head or muscle aches □ Fever □ Cough □ Tiredness □ Sore throat □ Conjunctivitis

□ Dyspnea □Nausea/Vomiting □ Diarrhea □ New loss of taste □ New loss of smell

B. ATTITUDES REGARDING COVID-19

The questions in this section are aimed at acquiring information about your attitudes regarding COVID-19

B1. How do you perceive your risk of developing COVID-19, on a 1 to 10 scale where 1 indicates low risk and 10 indicates high risk? (only if you have not been tested positive)

1 2 3 4 5 6 7 8 9 10

B2. For each statement answer whether you agree, are uncertain, or disagree

	Strongly disagree	Disagree	Uncertain	Agree	Strongly agree
It is important to get vaccinated against COVID-19					
COVID-19 is a serious disease					
I trust in the information received about COVID-19					

B3. For each statement answer whether or not are you concerned

	Extremely	Somewhat	Moderately	Slightly	Not at all
COVID-19 vaccine might not be safe					
COVID-19 vaccine might not be effective					

B4. Are you planning to have yourself vaccinated against COVID-19? (answer only if you have not contracted it)

□ Yes (more than one reason is allowed)	□ No (more than one reason is allowed)
REASONS	REASONS
□ If the vaccine is free of charge	□ If the vaccine is not free of charge
□ If physician recommends the vaccine	□ If physician does not recommend the vaccine
□ COVID-19 is a severe disease	□ COVID-19 is not a severe disease
□ Vaccine is safe	□ Vaccine is not safe
□ Vaccine is effective	□ Vaccine is not effective
□ I feel at risk	□ I do not feel at risk
□ Other reasons ________________________________	□ Other reasons ________________________________

C. ATTITUDE AND BEHAVIOR REGARDING INFLUENZA VACCINATION

The questions in this section are aimed at exploring your attitude and behavior regarding influenza vaccination

C1. Have you received the vaccination against seasonal influenza last year? □ No □ Yes

C2. Are you planning to have yourself vaccinated against seasonal influenza? (answer only if you have not received it)

□ Yes (more than one reason is allowed)	□ No (more than reason one is allowed)
REASONS	REASONS
□ If the vaccine is free of charge	□ If the vaccine is not free of charge
□ If physician recommends the vaccine	□ If physician does not recommend the vaccine
□ Seasonal influenza is a severe disease	□ Seasonal influenza is not a severe disease
□ Vaccine is safe	□ Vaccine is not safe
□ Vaccine is effective	□ Vaccine is not effective
□ I feel at risk	□ I do not feel at risk
□ Symptoms of COVID-19 and seasonal influenza can be very similar	□ Other reasons ________________________________
□ Other reasons ________________________________	

D. INFORMATION

The questions in this section are aimed to evaluate the sources from which you acquire information on COVID-19 vaccination

D1. From which of the following sources do you receive information about COVID-19 vaccination? (more than one answer is allowed)

□ None □ Internet □ Mass media □ Scientific journals □ Meetings/Conferences

D2. Do you feel you need more information about COVID-19 vaccination? □ No □ Yes
